# Laparoscopic Radiofrequency Thermal Ablation of Hepatocellular Carcinoma in Liver Cirrhosis Patients

**DOI:** 10.4021/gr490w

**Published:** 2012-11-20

**Authors:** Mohamed Ismail Seleem, Shawkat Shaker Gerges, Ashrif Elkhouly, Bahaa El-wakeel, Mohamed Hassany

**Affiliations:** a Department of Surgery and Tropical Medicine, National Hepatology and Tropical Medicine Research Institute, Cairo, Egypt

**Keywords:** Laparoscopy, HCC, RFA, IOLUS, Liver cirrhosis

## Abstract

**Background:**

Laparoscopic radiofrequency ablation (LRFA) for hepatocellular carcinoma (HCC) under guidance of intra-operative laparoscopic ultrasound (IOLUS) aiming of obtaining additional information for liver situation, better tumor staging and effective treatment of hepatic focal lesion (HFL) in patients with a difficult percutaneous approach.

**Methods:**

Between September 2010 and July 2012, 301 patients with HCC in liver cirrhosis were referred from HCC clinic at National Hepatology and Tropical Medicine Research Institute (NHTMRI). Twenty nine patients were submitted to LRFA with IOLUS guidance. Operation time, hospital stay, post procedure complication were recorded. Spiral CT scan one month postoperative was mandatory during follow up.

**Results:**

LRFA was completed in all patients. The IOLUS examination identified new HFL in three patients. A total of 32 lesions were treated. The mean operative time was 120 minutes; eight procedures were associated in six patients: cholecystectomy (6) and adhesiolysis (2). A complete tumor ablation was observed in all patients which were documented via spiral computed tomography (CT scan) one month after treatment.

**Conclusion:**

LRFA of HCC proved to be a safe and effective technique. IOLUS is superior on spiral CT scan in detection a small HCC.

## Introduction

Hepatocellular carcinoma, the most common primary liver cancer, occurs in 90% of the cases in patients with chronic liver disease (CLD) [1]. In recent years, its incidence has increased as consequence of chronic hepatitis C virus infections [2]. The optimal treatment for hepatocellular carcinoma is surgical resection. However, only a small percentage of patients are operative candidates. In 1868, the French scientist Jaques Arsen D’Arsonval described the principle of radiofrequency ablation demonstrating that an alternative electric current greater than 10 kHz could pass through living tissue without neuromuscular effect [3]. However, it was only in the late 1980s that new radiofrequency technology was developed which enabled ablation done within the body tissues [4]. Radiofrequency procedure can be performed through percutaneous, laparoscopic, thoracoscopic, and open approaches. Percutaneous radiofrequency ablation has been shown to be efficacious in the treatment of unresectable HCC, with complication rate around 2.1% [5]. Recent advances in laparoscopic ultrasound have greatly improved the accuracy in detecting intra-hepatic HCC nodules, many of which were missed by computed tomography [6]. Although, RFA and laparoscopy appear to be safe procedures with low rates of morbidity and mortality the indication should always be discussed in multidisciplinary meetings and the procedure should be performed only following guidance and adequate training.

## Patients and Methods

A total of 310 patients with HCC were treated using radiofrequency ablation (RFA) between September, 2010 and July, 2012 at National Hepatology and Tropical Medicine Research Institute-Cairo-Egypt. Twenty nine patients were contraindicated for percutaneous RFA, whom underwent laparoscopic RFA following the inclusion criteria (Table 1). History, physical examination, laboratory tests including complete blood picture, prothrombin time, liver functions, Hepatitis B and C profiles and serum alpha- fetoprotein (AFP) levels were obtained preoperatively. Abdominal ultrasound (US) and triphsic helical computed tomography (CT).

**Table 1:** Inclusion Criteria of RFA

Inclusion criteria
1. Contraindication of using percutaneous radiofrequency.
2. HCC near to vital organs such as diaphragm or gut.
3. Patients with Child-Pugh (A and B).
4. Patients who has an additional surgical indication such as, cholelithiasis, umbilical hernia.
5. Patients with hepatocellular carcinoma ≥ 5 cm in diameter.
6. Patients with American Society of Anesthesiologists (ASA) I, II, and III patients

### Laparoscopic RFA technique

**Figure 1:**
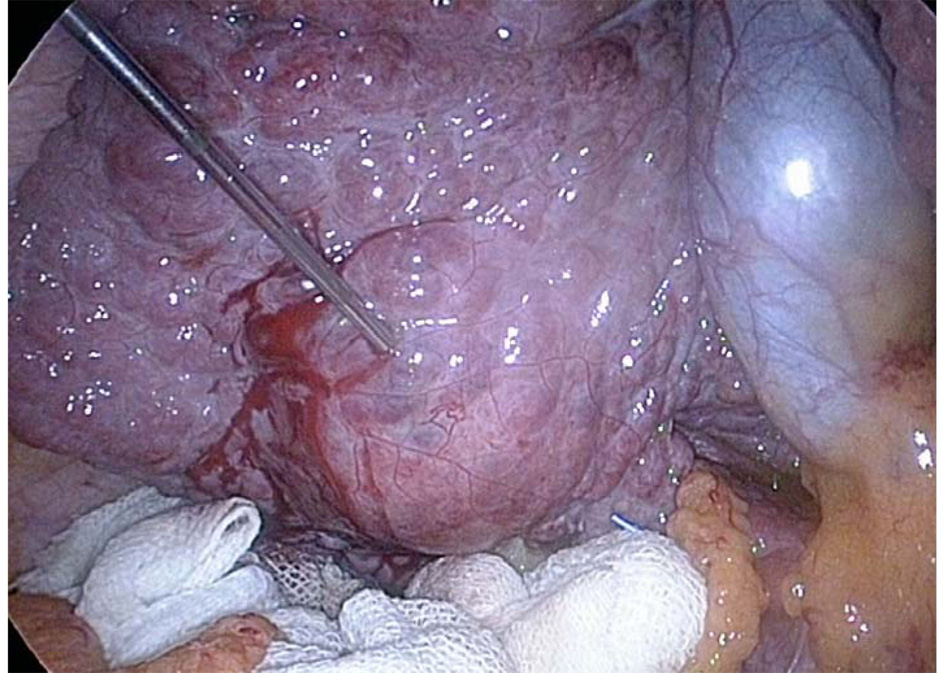
The colon was protected with abdominal gauze soaked in saline solution.

**Figure 2:**
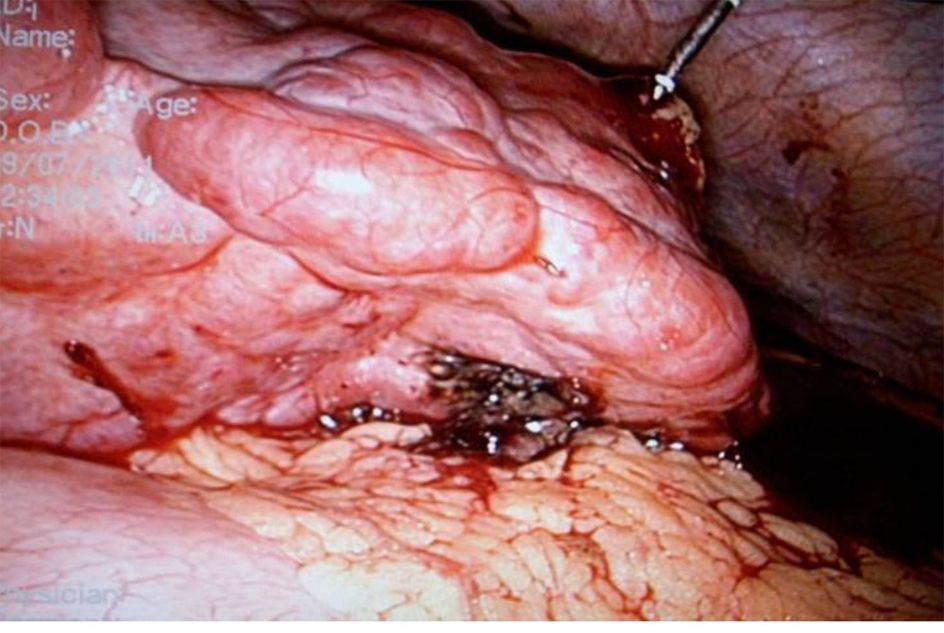
The stomach was protected by omentum.

**Figure 3:**
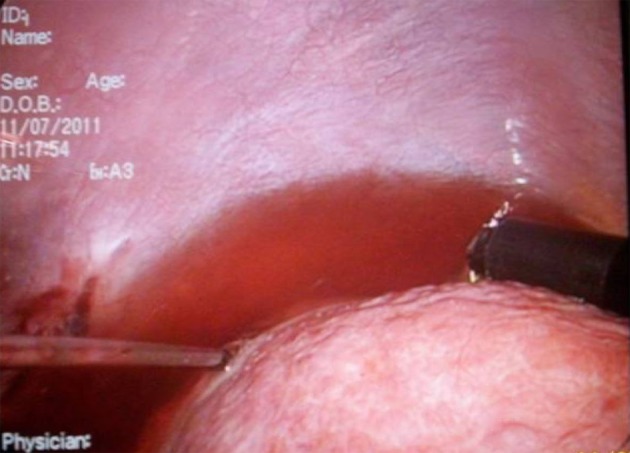
Creation of ascites by instillation of Dextrose 5% to protect the diaphragm.

**Figure 4:**
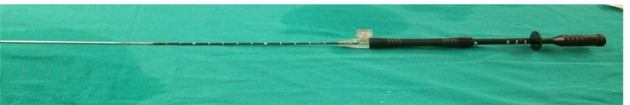
Semi-flax retractable multi-pronged curved electrode needle.

The procedure was performed with the patient under endotracheal general anaesthesia and placed in a supine position on operating table. The surgeon stood on the left side of the patient and the first assistant on the opposite side. The monitors were placed at the head of the table, through an infra-umbilical incision, carbon dioxide pneumoperitoneum was carried out using Veress needle. In patients with prior open abdominal operations, the abdominal cavity was entered by using the open technique. Laparoscopic exploration was performed with a zero degree laparoscope. Another 10 mm port was placed according to the location of the HCC, with addition of another 5 mm port. Laparoscopic ultrasound carried out for whole liver followed by localization of HCC. The vital organs near HCC such as stomach, colon or diaphragm were protected by abdominal gauze soaked in saline solution, introduced through the 10 mm port (Fig. 1), or omentim (Fig. 2), and in some cases creation of ascites by installation of 5% dextrose to displace or protect adjacent viscera was indicated (Fig. 3). RFA was performed under ultrasonography guidance, utilizing a generator providing 460 kHz alternating current and a semi-flax retractable multi-pronged curved electrode-needle (Fig. 4) (RITA medical system, Mountain View, California). The average target temperature was set at 100 °C to 110 °C, and ablation was continued for 25 - 30 minutes depending on the desired ablation size (3 - 5 cm in diameter). The process was monitored by real- time ultrasound to ensure 1 cm margins. The drain was left behind. Drains were removed within 24 - 48 hours.

Operative time, complication and hospital stays were recorded. Triphasic CT scan was obtained one month post-operatively and abdominal ultrasound and AFP were obtained every three months during follow up period. Whenever AFP re-elevated, further imaging studies such as Triphasic CT scan was performed. Local recurrence was defined as tumor recurred at the treated site. However, new tumor which appeared in different hepatic parenchyma was defined as new HCC. For these recurrences or new HCC RFA was considered and performed either percuteanous or laparoscopic as indicated.

## Results

Laparoscopic RFA was performed in 29 patients. The mean age was 60 years (48 - 74 years). There were 24 males and 5 females patients. All patients were hepatitis C positive. The mean operative time was 120 minutes (80 - 180 minutes). The mean lesion size was 3.5 cm (1.5 - 5 cm). The distributions of lesion site are shown in (Table 2). The mean hospital stay was 1.5 days (1 - 2 days). There was no mortality (within 30 days of surgery) in this series. Complications were noted as the following, nine patients underwent refractory ascites, one case has local recurrence that underwent transarterial chemoembolization and one case has a new intrahepatic HCC who underwent a second attempted of laparoscopic RFA.

**Table 2:** Distributions of Lesion Site

Items	Results
Mean age	60 years
Mean operative time	120 minutes
Mean hospital stay	1.5 days
Mean lesion size	3.5 cm
Lesion site	Segment-II: 5 cases Segment-III: 3 cases Segment-IV: 2 cases Segment-V: 2 cases Segment-VI: 4 cases Segment-VII: 4 cases + one new lesion Segment-VIII: 9 cases + two new lesions
Complications	Fever: 5 patients Ascites: 9 patients Recurrence: one patient

## Discussion

Hepatocellular carcinoma is the fifth most common cancer worldwide. The treatment of HCC in patients with chronic liver disease is a major challenge. Although there is a certain agreement about avoiding surgery in patients with more advanced chronic liver disease, which treatment has to be offered to patients with a relatively preserved liver function is still a matter for debate [7]. However, only selected patients are suitable for surgical resection because of advanced tumors, major vascular invasion, multifocal tumors, poor hepatic reserve or extra hepatic disease. With the intention of avoiding the risk of hepatic failure that can follow hepatic resection in such patients, percutaneous ablative treatments have been proposed, of which radiofrequency ablation is progressively gaining consensus due to the efficacy, tolerability and low-risk of procedure [8] Therefore, the need of alternative treatments of HCC local ablative therapies including percutaneous ethanol injection, acetic acid injection, cryotherapy, microwave coagulation, laser and radiofrequency ablation, have been investigated. Currently, the last has been most enthusiastically utilized. Rossi et al [9] the first described RFA of human liver tumors in a large study in the early 1990s. While the earliest recorded use of heat to treat tumors dates back to Egyptian and early Greek medical descriptions [10]. Bleeding, liver abscesses and colonic perforation were the most frequently reported complications, and less-frequent complications were bilomas, biliary strictures and haemothorax [11]. Laparoscopic radiofrequency ablation was able to avoid two of the most frequently complication, bleeding, which could be controlled under supervision during laparoscopic technique as well as colonic perforation by different ways of protection as mention before. Haemothorax is one of less frequent complication could be avoided in laparoscopic radiofrequency ablation by using a technique of creation ascites as described in this series. However, the other complications were not detected in this series during follow up period.

A laparoscopic approach offers the advantages of laparoscopic ultrasonography, which provides better resolution of the number and location of liver tumors, and a survey of the peritoneal cavity to exclude the presence of extra-hepatic disease. The laparoscopic ultrasound permits more precise positioning of the radiofrequency needle multiple array near major blood vessels. In this series, we use a laparoscopic approach for patients according inclusion criteria. In our series, laparoscopic ultrasound was able to detect a new lesion in three patients which treated simultaneously. Laparoscopic RFA has been recommended to treat patients with an additional surgical problems such gall bladder stones. Six patients had laparoscopic cholecystectomy in additional to RFA. Ultimately, the best management of HCC would involve prevention of viral hepatitis, early detection and liver transplant. However, laparoscopic RFA is much less invasive, involves a short hospital stay, and an extremely low mortality associated with the procedure. We conclude that laparoscopic radiofrequency ablation of HCC is a safe, feasible treatment modality to achieve good tumor ablation with great accuracy under laparoscopic ultrasound guidance permitting to detect new lesion missed at preoperative imaging.
